# Localized Down-regulation of P-glycoprotein by Focused Ultrasound and Microbubbles induced Blood-Brain Barrier Disruption in Rat Brain

**DOI:** 10.1038/srep31201

**Published:** 2016-08-11

**Authors:** HongSeok Cho, Hwa-Youn Lee, Mun Han, Jong-ryul Choi, Sanghyun Ahn, Taekwan Lee, Yongmin Chang, Juyoung Park

**Affiliations:** 1 Daegu-Gyeongbuk Medical Innovation Foundation, Medical Device Development Center, Daegu, 41061, South Korea; 2 Kyungpook National University, Department of Medical & Biological Engineering, Daegu, 41566, South Korea; 3 Daegu-Gyeongbuk Medical Innovation Foundation, Laboratory Animal Center, Daegu, 41061, South Korea

## Abstract

Multi-drug resistant efflux transporters found in Blood-Brain Barrier (BBB) acts as a functional barrier, by pumping out most of the drugs into the blood. Previous studies showed focused ultrasound (FUS) induced microbubble oscillation can disrupt the BBB by loosening the tight junctions in the brain endothelial cells; however, no study was performed to investigate its impact on the functional barrier of the BBB. In this study, the BBB in rat brains were disrupted using the MRI guided FUS and microbubbles. The immunofluorescence study evaluated the expression of the P-glycoprotein (P-gp), the most dominant multi-drug resistant protein found in the BBB. Intensity of the P-gp expression at the BBB disruption (BBBD) regions was significantly reduced (63.2 ± 18.4%) compared to the control area. The magnitude of the BBBD and the level of the P-gp down-regulation were significantly correlated. Both the immunofluorescence and histologic analysis at the BBBD regions revealed no apparent damage in the brain endothelial cells. The results demonstrate that the FUS and microbubbles can induce a localized down-regulation of P-gp expression in rat brain. The study suggests a clinically translation of this method to treat neural diseases through targeted delivery of the wide ranges of brain disorder related drugs.

Many effective therapeutic drugs have been developed to treat central nervous system (CNS) related diseases. However, the effectiveness of most of those drugs had been hindered by the presence of a barrier between the CNS and the circulating blood; the blood-brain barrier (BBB). The BBB is a physical and metabolic barrier, which serves to regulate the flow of essential components into and out of the central nervous system (CNS) and to prevent the influence of toxic compounds and pathogens to the CNS^1^,^2^,^3^. The BBB consists of endothelial cells connected by junction complex in which tight junction (TJ) acts as a physical barrier^4^. The BBB remains one of the greatest challenges for the discovery and development of treatments for CNS disorders, which to this day remains one of the riskiest disease areas in terms of clinical success rates.

Different strategies have been used to overcome the BBB, including direct drug injection/infusion^5^, trans-arterial infusion of agents such as mannitol for a transiently BBB disruption^6^,^7^, or by developing new drug formulation that can cross the BBB^8^,^9^. However, these methods have not been shown to be reliable or effective in actual clinical trials, because they all have been either invasive, non-targeted, or required the formulation of new drugs^10^.

Recently, focused ultrasound (FUS) combined with microbubbles has emerged as a promising method to temporarily permeabilize the BBB in a targeted region enabling a localized drug delivery for brain tumors and other disorders of the CNS^11^,^12^. This method utilizes the mechanical effect of microbubble oscillations induced by the focused ultrasound for a transient disruption or loosening of TJ in the BBB^2^,^13^. This transient BBB opening lasts for 4 to 6 hours providing a good temporal window for drug delivery^14^. Promising results are reported from experiments on macaques^15^,^16^ and human clinical trials are currently ongoing in order to validate the effectiveness and the safety of this method.

Even with the disruption of the physical barrier of the BBB, there is still another barrier which impedes the effective delivery of the drugs into the brain. ATP-binding cassette (ABC) efflux transporters such as P-glycoprotein (P-gp) in the brain the endothelial cells serve as a functional barrier, preventing the brain uptake of harmful compounds^17^,^18^. These multi-drug resistance active transport proteins localized in the membrane of brain capillary endothelial cells actively pumps out most of the drugs, reducing the drug retention and accumulation in brain tissue^19^,^20^. Together with the physical barrier, this functional barrier of the BBB becomes a great challenge in treatments of brain related diseases, because these barriers precludes the effective passing and retention of most chemotherapeutics from the blood circulation to the brain tissue^21^,^22^.

The purpose of this work was to investigate the impact of FUS and microbubbles on the functional barrier of the BBB through an *in-vivo* experiment on rat brains. In this work, we limited ourselves to focus on the expression of P-gp, the most known multi-drug resistance transporter that is expressed at the brain endothelial cells^23^. The results from this study may contribute to clinical translation of the technology for treatment of neural diseases by enhanced and targeted delivery of the wide ranges of CNS disorder related drugs.

## Results

### BBB disruption and P-gp expression

From a pilot study, successful BBB disruptions were confirmed through enhanced the intensity of a MR contrast agent in T1-weighted MR images. The range of feasible acoustic pressure for BBB disruption was found at range of 0.6 MPa–0.65 MPa peak negative pressure (see Supplementary Fig. S1).

**Figure 1:**
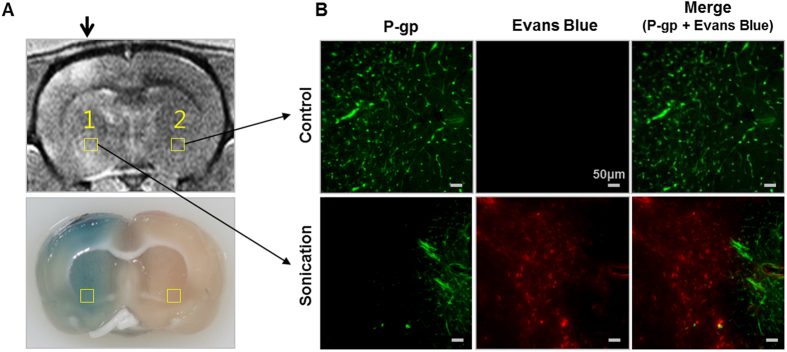
Representative data obtained from a rat brain comparing a sonication region and a control region after focused ultrasound and microbubbles treatment. (**A**) Top: Contrast-enhanced T1-weighted MR image of a rat brain after sonication. The sonicated direction is indicated by the black arrow. Location 1 and 2 is selected for fluorescence microscopy for P-gp expression and Evans Blue. Bottom: The corresponding tissue slice of the rat brain. The BBB disruption region is indicated by Evans Blue dye. (**B**) Top: Fluorescence images of the control region showing P-gp expression intensity and Evans Blue intensity, respectively. Bottom: Fluorescence images of the sonicated region.

The FUS and microbubbles successfully disrupted BBB of a rat brain as shown in Fig. 1. Contrast enhancement in a T1-weighted MR image at the sonicated region indicates successful delivery of a MR contrast agent through the BBB. Blue dyed area in the extracted perfused brain slice confirms the Evans Blue penetration through the BBB (Fig. 1A). A Fluorescence microscopy image (Fig. 1B) at the control region shows active P-gp expression (green) and no sign of Evans Blue (red). Whereas for the BBB disruption region, P-gp expression was partially reduced and the down-regulated area coincides with the area dyed with Evans Blue.

**Figure 2:**
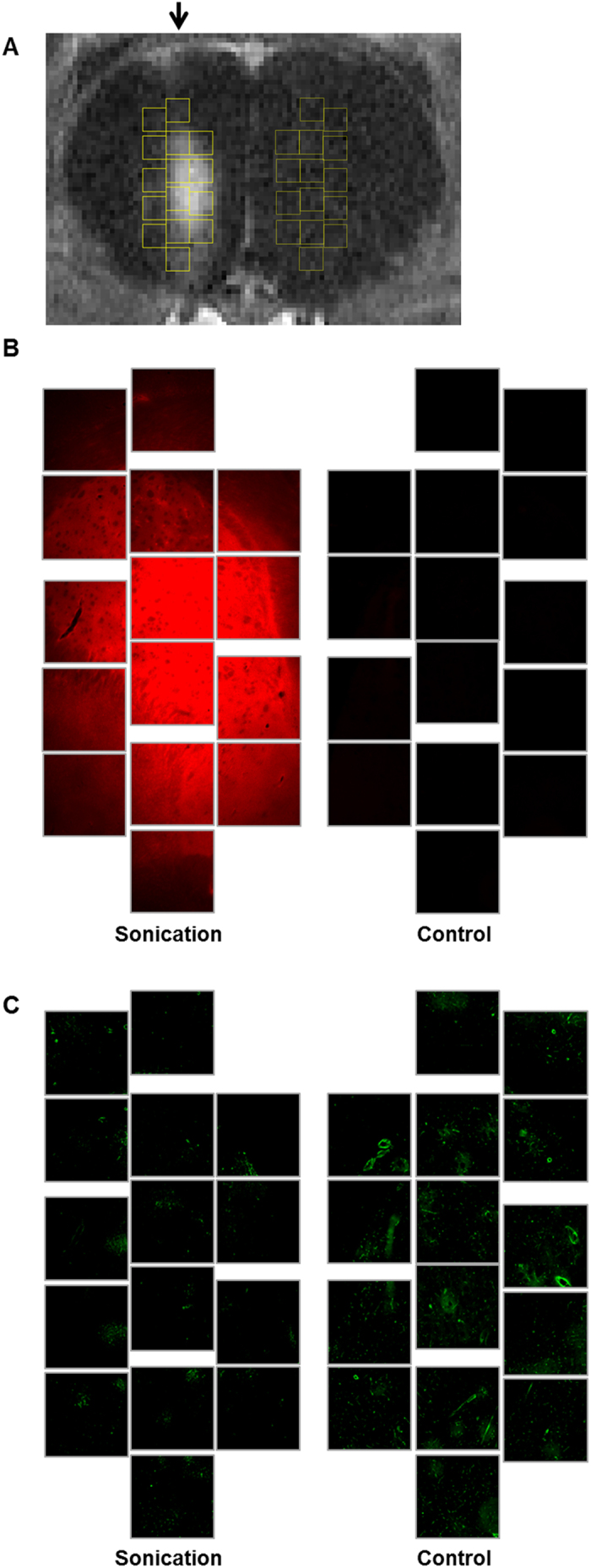
An MR and fluorescence images of a rat brain after a sonication. (**A**) Contrast-enhanced T1-weighted MR image of a rat brain after sonication. The sonicated direction is indicated by the black arrow. Yellow squares indicate 15 selected areas over sonication region and control region for fluorescence analysis of P-gp expression and Evans Blue. (**B**) Immunofluorescence images of the selected areas at the sonication and control regions. The red fluorescent indicates BBB disruption with Evans Blue penetration. (**C**) Immunofluorescence images of the selected areas with green fluorescent indicating P-gp expression.

This localized down-regulation of P-gp expression resulted in all three rats. Areas were selected over the BBB disruption region from contrast enhanced T1-weighted MR images. Areas at corresponding opposite side of the brain were used as controls. Fluorescence images at the selected areas were analyzed for the Evans Blue and the P-gp expression. Figure 2 shows the MR and the fluorescence images at the selected areas.15 areas over sonication regions and corresponding control regions (yellow squares) were selected in T1-weighted MR image in one of the three rats (Fig. 2A). The contrast enhanced areas in the MR image matched with the Evans Blue dyed areas in the fluorescent image (Fig. 2B). In the same areas, the P-gp expression was reduced compared to the control areas (Fig. 2C).

**Figure 3:**
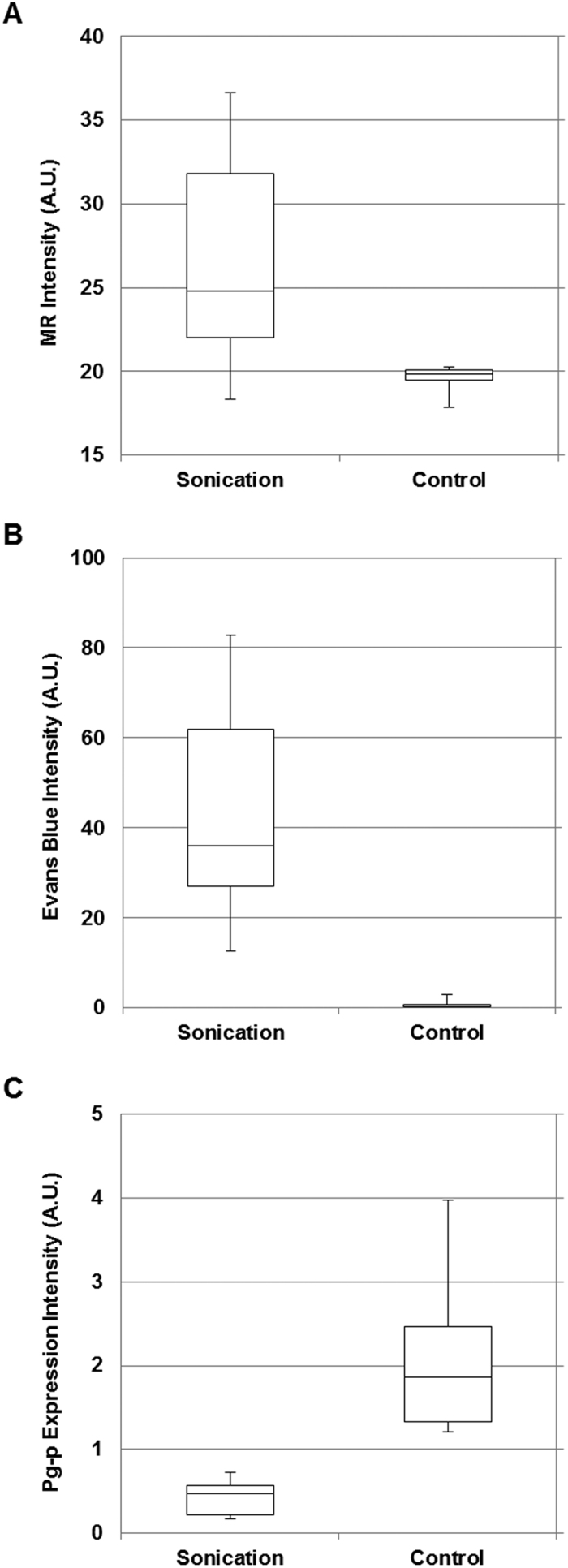
Boxplot comparing sonicated region and control region in a total 31 selected areas from three rats. (**A**) The MR contrast intensity at the sonication region was compared to the control region. (**B**) The Evans Blue fluorescence intensity at the sonication region and the control region were compared. (**C**) The P-gp expression fluorescence intensity was compared between the sonication and the control region.

For a detailed analysis, the contrast (MR) and the fluorescence (Evans Blue and P-gp) intensities between the sonicated and the control areas at a total of 31 selected areas in all three rats were compared statistically (Fig. 3). In total 31 selected areas in the treatment group animals (n = 3), the mean contrast enhancement of MR intensity was 27.5% ± 4.5 (standard error of the mean). The mean increase of Evans Blue fluorescent intensity at the sonication region was 32.4 (A.U) ± 3.8 compared to the control region. The P-gp expression intensity at the sonication region was reduced by an average of 63.2% ± 4.2 compared to the control region. The differences at MR intensity, Evans Blue fluorescent intensity and P-gp expression intensity were statistically significant in the total 31 selected areas (p < 0.001).

### Correlation between BBB opening and P-gp expression

**Figure 4:**
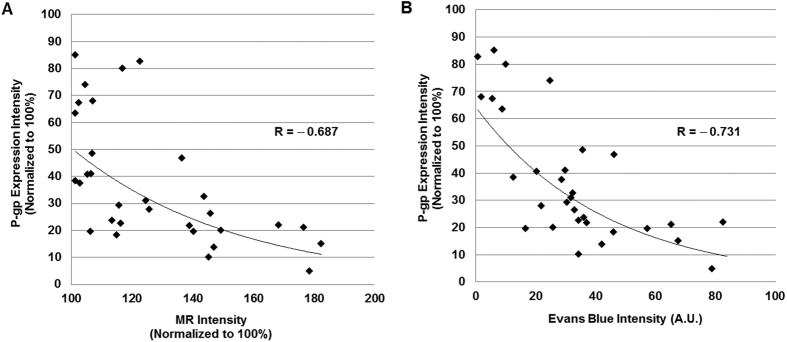
Correlation between P-gp expression and BBB opening. (**A**) Relationship between MR contrast intensity and P-gp expression (**B**) Relationship between Evans Blue intensity and P-gp expression: The correlation between the magnitude of BBB disruption and the down-regulation of P-gp expression were significant. (R = −0.687, p < 0.001, n = 31; R = −0.731, p < 0.001, n = 31, respectively).

The gradient of the acoustic pressure field around the focal region induced different magnitude of BBB openings at the sonication region (See Supplementary Fig. S2). In order to assess the relationship between magnitude of BBB opening and the P-gp expression, the 31 areas selected over sonication regions and corresponding control regions of the treatment group animals were analyzed for correlation. Figure 4A shows correlation between MR contrast intensity and P-gp expression at the sonication region both normalized to 100% compared to the control region. The correlation between the BBB disruption magnitude (MR and Evans Blue intensities) and the level P-gp down-regulation was significant (p < 0.001). (Figure 4). The modest difference in correlation trend between Fig. 4A,B is addressed in discussion section.

### Safety analysis

**Figure 5:**
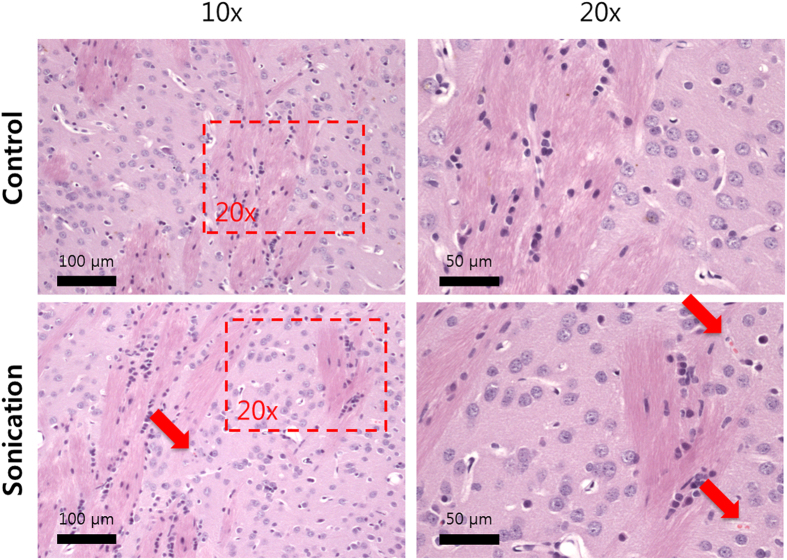
Histology slices of the sonicated rat brains. Images of sonication location show normal tissue matrix with some extravasations is visible (Hematoxylin-eosin stain).

H&E histologic analysis of the acute specimens revealed that sonications resulted in either no apparent change in the tissue or in some extravasated red blood cells as shown in Fig. 5. Only in a few spot there were regions with a small amount of red blood cells indicated by red arrows in Fig. 5, without visible large vessel rupture.

In addition, any presence of adverse impacts of FUS and microbubbles on brain endothelial cells functions was observed by comparing RECA-1 expression at the BBB disruption region and the control region. The RECA-1 expression was not reduced at the sonication region compared to the control region (see Supplementary Fig. S3), demonstrating the resulted down-regulation of P-gp expression was not stimulated by endothelial cell’s functional impairment.

## Discussion

The overall results of our study demonstrate the inhibition of the P-gp by the FUS and microbubbles. Also, there was a significant relationship between the level of the down-regulation of the P-gp expression and the magnitude of the BBB opening. The BBB disruptions and the down-regulations of the P-gp expression involved no damage to the tissue and the functions of endothelial cells of the brain. These results suggest direct translation of the technique to medicine. P-gp substrates include recognized anticancer drugs such as Doxorubicine, daunorubicine, vinblastine, vincristine, etoposide, teniposide^1^,^17^,^18^. Studies have shown that the brain penetration of P-gp substrate drugs can increase up to 10- to 100-fold in the absence of P-gp in the BBB in *mdr1a* knockout mice^17^. Inhibition of P-gp by FUS and microbubbles may allow use of these well-known drugs to prolong retention in brain tissue after across the BBB. Similarly, enhanced drug delivery and drug retention by down-regulating P-gp induced by FUS and microbubbles may lead to reduction of amount and frequency of the drug treatment, minimizing side effects on the patients. In addition, FUS and microbubbles could modulate P-gp effect on the localized brain area, which further increases the effectiveness of the drug and minimizes the toxic effect of the drug to the surrounding tissue.

In brain disease such as epilepsy, depression, or schizophrenia patients are resistant to current medications despite adequate choice of therapeutic drugs at maximum tolerated doses, mainly due to the low retention rate of the drug by the multi-drug transporters at the BBB^24^,^25^,^26^. Moreover the P-gp and multi-drug resistance proteins are widely found in tumor, which are resistance to diverse chemotherapeutic agents^27^. In order to overcome these barriers pharmaceutical industry has developed a large number of P-gp and multi-drug resistant protein inhibitors. The use of cerebral P-gp inhibitors significantly increased the brain concentration of various drugs by down-regulation of P-gp in the BBB^28^,^29^. However, modulation of P-gp in clinical oncology has had limited success as yet^27^,^30^. The inhibitors developed lacked specificity, required high doses and were associated with unacceptable toxicities^31^. In addition, studies have found that the drugs that are normally well tolerated may become neurotoxic in the absence of active transporters at the BBB^17^,^19^. Therefore, a local and transient inhibition, short-term administration of inhibitors may be useful strategy. Our results suggest that the FUS and microbubbles may enhance drug delivery and retention by local and reliable P-gp inhibition without toxicity. There were a few attempts to regulate the P-gp by FUS in *in-vitro* cell studies^32^,^33^,^34^, However, this work is a first to observe the phenomenon *in-vivo* in the BBB.

Past studies have shown that the predictable oscillation of microbubbles, also known as ‘stable’ cavitation, generates mechanical stress and shear stress to the blood vessel wall^35^,^36^,^37^. Results from past studies hypothesized that these mechanical stresses may impact mechanosensitive proteins in the endothelial cells^38^. For example, reduction of tight junction proteins such as occludin, claudin-5 after FUS treatment has been detected using electron microscopy^13^,^39^. Also a gap junction protein, Connexin 36 was down regulated after FUS treatment with microbubbles^40^. Low P-gp expression observed in arterioles of rat brain where shear stress is high also supports the hypothesis^33^. In addition, there was a recent study showing that low intensity ultrasound down regulated the expression of P-gp and multidrug resistance protein (MRP) 1 in rat brain glioma^41^. These results suggest that the stable oscillation of microbubbles may regulate ion channel in the BBB causing up or down regulation mechanosensitive proteins. Further study on the mechanism of the P-gp regulation by FUS and microbubble is needed.

Recent applications of FUS combined with microbubbles as a therapeutic method have shown several promising biological effects, including BBB disruption accompanied by reversible changes in neuron responses^42^, diminution of amyloid-β in an Alzheimer’s disease^43^, and enhanced drug delivery and treatment of brain tumors^44^,^45^,^46^. However, these previous studies only suggested FUS and microbubbles’ impact on the tight junction. Our study suggests that in those previous results, there have been the impact on down-regulation of P-gp and potentially other multi-drug resistant efflux transporters.

There are few limitations in this study. First, no actual P-gp substrate drug was administered in this study. Our past work showed FUS and microbubbles not only enhanced the delivery of the chemotherapy agent doxorubicin to the rat brain tumors, but also retained drug concentrations in sonicated tumors for at least 24 hours^47^. However, in order to confirm the promising result of down-regulated P-gp, future studies with the administration of actual P-gp substrate drugs are needed. Also, the future work may include analysis on the change in P-gp expression in tumor models due to FUS and microbubbles.

In this work, we only observed a down-regulation of P-gp expression at 24 hours after the BBB disruption. In the correlation analysis, the contrast enhanced MR image was taken immediately after the FUS treatment with microbubbles, while the P-gp and the Evans Blue fluorescence intensities were measured on brains that were perfused and fixed 24 hours after the FUS treatment. In Fig. 4A, there is a larger variation in the P-gp expression intensity for the areas near the periphery of focal region compared to that of the Fig. 4B. The temporal gap between the MR data and the P-gp fluorescence data may have caused this variation. In order for this method to be applied clinically, an effective temporal window of the P-gp down-regulation due to FUS and microbubble is important. Also, the P-gp kinetics may be strongly influenced by the kind and the concentration of drugs when combined with FUS and microbubbles. Therefore, additional studies are needed in order to observe the change in P-gp expression over time after the BBB disruption.

Lastly, even though the P-gp is a most known active efflux transporter found in the BBB, there are other multi-drug efflux transporters such as breast cancer resistance protein (BCRP) and members of the multi-drug resistance protein (MRP) family, and their substrates vary. Our study focused in P-gp, but future work needs to investigate the other transporters found in BBB.

## Methods

### Animals

All experiments were done in accordance with procedures approved by the Daegu-Gyeongbuk Medical Innovation Foundation (DGMIF) Institutional Animal Care and Use Committee (IACUC). All procedures and animals handlings were performed following the ethical guidelines for animal studies. The animals were anesthetized during all procedures and were constantly monitored throughout the experiment. No pain or suffering was evident as a result of the procedure. A total of 25 male Sprague-Dawley rats (250–350 g weight, Orient Bio Inc., Seongnam, Korea) were used for this study. First 18 rats were used in pilot study to define experimental settings for stable BBB disruption (BBBD). Three rats were used for the *in-vivo* experiment for the P-gp evaluation. Remaining four rats were used for RECA-1 fluorescence analysis and Hematoxylin and Eosin (H&E) histology for assessment of any damage in rat brain endothelial cell.

### BBB Disruption System Setup

**Figure 6:**
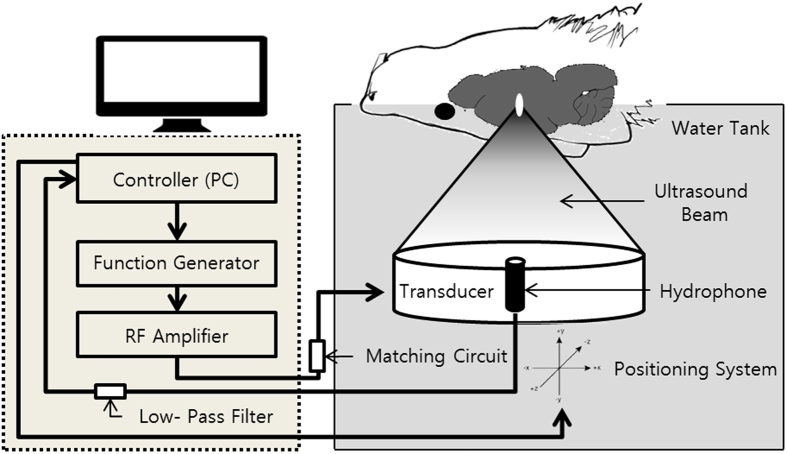
Experimental setup for acoustic emission controlled BBB disruption (RK-100, FUS Instruments, Toronto, Canada). The animal is supine and the head is submerged in water tank. The focal area is targeted with MR image guidance and PC-controlled positioning system. The transducer output is feedback controlled by monitoring acoustic emission received through hydrophone located at the center of the transducer.

A preclinical MRI-guided Focused Ultrasound (MRgFUS) system (RK-100, FUS Instruments, Toronto, Canada) was used to sonicate rat brains for BBB disruption. The system schematic is shown in Fig. 6. The system has an air-backed, single-element, spherically-curved, piezoelectric transducer (FUS Instruments, Toronto, Canada) with a diameter of 75 mm, a radius of curvature of 60 mm, and a resonant frequency of 1 MHz for generation of the ultrasound field. The free field ultrasound pressure distribution at focal region was measured in Acoustic Intensity Measurement System (AIMS III, ONDA, Sunnyvale, CA, USA) with a hydrophone (HGL-400, ONDA, Sunnyvale, CA, USA). The ultrasound pressure distribution was measured with and without a rat skullcap in place in order to estimate the *in situ* pressure accounting for the attenuation due to the skull. (See details in Supplementary Fig. S4). The transducer was driven by a signal generated by an arbitrary waveform generator (33220A, Agilent, Santa Clara, CA, USA) and amplified with an RF power amplifier (4010L, E&I, Rochester, NY, USA). The transducer was submerged in a water tank filled with degassed water, and the animal was placed supine on MR compatible animal bed with its head partially submerged in water. A horizontal bore 9.4 preclinical MRI (BioSpec 94/20 USR, Bruker, Billerica, MA, USA) was utilized as image guidance for the FUS system. MR images were used to target specific region of the rat brain for sonications. Images taken from the preclinical MRI was transferred to the MRgFUS system and then the coordinates between the two systems were put in sync.

### BBBD Experiment

In prior to experiment on P-gp, a pilot study was performed with 18 rats. The pilot study aimed to refine the experimental settings, ultrasound parameters and microbubble conditions for safe and repeatable BBB disruptions. During sonications, the acoustic emission signal from FUS induced microbubble oscillation was observed through a PZT hydrophone located at the center of the transducer. A range of sufficient and adequate ultrasound intensity was found by monitoring the magnitude of subharmonic signal intensity (see Supplementary Fig. S1). This method was used in past studies to find ultrasound pressure for robust and safe disruption of the BBB^48^,^49^. The quality of BBB disruptions were evaluated through MR imaging. The MR parameters found from the pilot study were used for *in-vivo* experiment of P-gp expression analysis.

For *in-vivo* experiment, rats were anesthetized with mixture of Zoletil 25 mg/kg (Virbac Laboratories, France) and Rumpun 4.6 mg/kg (Bayer, Leverkusen, Germany) intramuscularly. The hair on their heads was removed using a shaving razor and hair removal cream. The animals were placed supine on a MR-compatible animal bed as described in Fig. 6. T1-weight MR images were taken for the image guided treatment planning. Sonication targets were selected over the MR image of a rat brain.

A range of 0.6–0.65 MPa peak negative acoustic pressure at focal region was used for the experiment. The range corresponds to approximately 0.3–0.35 MPa peak pressure *in vivo* (See details in Supplementary Fig. S4). This ultrasound energy was delivered in pulsed sonications consisted of 10 msec bursts at a pulse repetition frequency of 1 Hz for 120 seconds (duty cycle: 1%). The microbubble contrast agent (0.02 mL/kg, Definity; Lantheus Medical Imaging, North Billerica, MA, USA) was diluted 1:50 in normal saline and injected through a tail vein catheter using an automated syringe pump (Pump 11, Harvard Apparatus, Holliston, MA, USA). Microbubbles were infused over 90 seconds. The sonication was started 30 seconds after the microbubble injection was initiated, in order to ensure that the focused ultrasound was treated when the circulating microbubbles have fully reached the targeted brain.

After the sonication, T1-weight MR images were taken with and without the 0.2 mM/kg gadolinium-based contrast (Magnavist; Berlex Laboratory, Wayne, NJ, USA), respectively. 0.5 ml Evans Blue (Sigma-Aldrich, St. Louis, MO, USA) was injected intravenously in order to determine the BBB disruption regions through both histologic and immunofluorescence analysis^50^. All brains were perfused and fixed via transcardial perfusion (0.9% NaCl, 100 mL; 10% buffered formalin phosphate, 250 mL) 24 hours after the sonications. Following perfusion, all brains were extracted and prepared for the assessment of effectiveness of the BBB disruption and the P-gp expression through fluorescence imaging.

In order to assess any presence of damage in brain tissue and endothelial cells due to BBB disruptions, additional 4 rats sonicated. The procedure and protocol of the sonication was identical to the *in-vivo* experiment for P-gp evaluation. Following perfusion, all brains were extracted and two of them were prepared for RECA-1 fluorescence imaging and the remaining two were prepared for a histologic analysis. The details are described in the following sections.

### MR Imaging

Imaging was performed using the 9.4T preclinical MRI system described above. An 86-mm inner diameter volume coil was used for RF transmission and signal reception. T2-weighted imaging was used to select the sonication targets and detection of edema. 2D RARE pulse sequence was used for acquisition of T1-weighted images for the evaluation of BBB disruption (see Supplementary Table S1 for detailed MR parameters).

### Fluorescence Imaging

Rats were sacrificed 24 hours after sonication for determination of P-gp, Evans Blue and RECA-1 expression. P-gp fluorescence image was used to evaluate the change in the P-gp expression due to FUS and microbubbles-induced BBBD. The Evans Blue fluorescence image was used to describe the area of successful BBB disruptions. The RECA-1 expression was used to validate the presence of the functional damage on the brain endothelial cells^51^.

Rats were perfused as previously described^52^ and 50 μm thick brain slices were cut on a cryostat. The slices were pretreated with mixture of ethanol and acetic acid (2/1) for 10 mins at −20 degree and rinsed in 0.1% Triton X100 in phosphate-buffered saline (PBS). Next, the slices were permeabilized in 0.5% Triton X100 for 30 mins and rinsed in PBS. Free aldehydes were then quenched by incubation in 1% NaBH4 in PBS for 20 mins at room temperature. To block nonspecific antibody binding, slices were incubated with PBS containing 1% bovine serum albumin (BSA), 10% goat serum, and 0.1% Triton X100. Slices were washed three times in phosphate buffered saline with tween (PBST) for 10 mins and incubated for 16 hours at 4 degree in mouse anti-P-gp (C219, Enzo, New York, USA) or anti-RECA-1 (ab9774, Abcam, Cambridge, UK) 1:50 diluted in PBST. After thorough washing in PBST, slices were incubated in Alexa Fluor 488 goat anti-mouse antibody (Invitrogen, Thermo Fisher Scientific, Waltham, MA, USA) (1:300 in PBST) for 2 hours at Room temperature. Slices were mounted with Fluorescence mounting medium (S3023, Dako, Glostrup, Denmark)^33^.

The fluorescence CCD (iXon, Andor Technology, Belfast, United Kingdom) images were recorded on a confocal fluorescence microscope (Eclipse Ti-E, Nikon, Japan with X-light, Crest Optics, Italy) equipped with a 10 X objective. Two channels were acquired sequentially with the following excitation and emission parameters: 488 nm, 500 to 540 nm for P-gp and RECA-1; 550 nm, 590 to 620 nm for Evans Blue, respectively. To remove background from the image, we performed background subtraction on Metamorph software (Molecular Devices, Sunnyvale, CA, USA).

### Histology

To evaluate the histological effects of the ultrasound protocol used in this study, the extracted brains were embedded in paraffin, and serially sectioned at 5 μm sections in the axial plane (perpendicular to the direction of ultrasound beam propagation). Every 50^th^ section (250 μm apart) was stained with H&E. The author who evaluated the histology was blind to the FUS exposure parameters. The images were recorded on an upright microscope (ECLIPSE Ni-U, Nikon, Japan) using 10 X and 20X objectives.

### Statistical analysis

The MR contrast intensity, Evans Blue and P-gp fluorescence intensities for the sonicated and contralateral (control) regions were compared using a two-tailed paired student’s t test. The correlation analysis between P-gp down-regulation, MR contrast enhancement and Evans Blue fluorescence intensity were evaluated using two-tailed Pearson correlation. For all statistical analysis, values of p < 0.05 were considered statistically significant.

## Additional Information

**How to cite this article**: Cho, H. *et al*. Localized Down-regulation of P-glycoprotein by Focused Ultrasound and Microbubbles induced Blood-Brain Barrier Disruption in Rat Brain. *Sci. Rep.* **6**, 31201; doi: 10.1038/srep31201 (2016).

## Supplementary Material

Supplementary Information
