# Supplementary material for: Localized Down-regulation of P-glycoprotein by Focused Ultrasound and Microbubbles induced Blood-Brain Barrier Disruption in Rat Brain
Source: Sci Rep. 2016 Aug 11;6:31201. doi: 10.1038/srep31201 (PMC4980618; doi:10.1038/srep31201)
Supplement: Supplementary Information [file srep31201-s1.pdf]

# Localized Down-regulation of P-glycoprotein by Focused Ultrasound and Microbubbles induced Blood-Brain Barrier Disruption in Rat Brain

**HongSeok Cho, Hwa-Youn Lee, Mun Han, Jong-ryul Choi, Sanghyun Ahn, Taekwan Lee, Yongmin Chang, Juyoung Park**

## Supplementary Materials

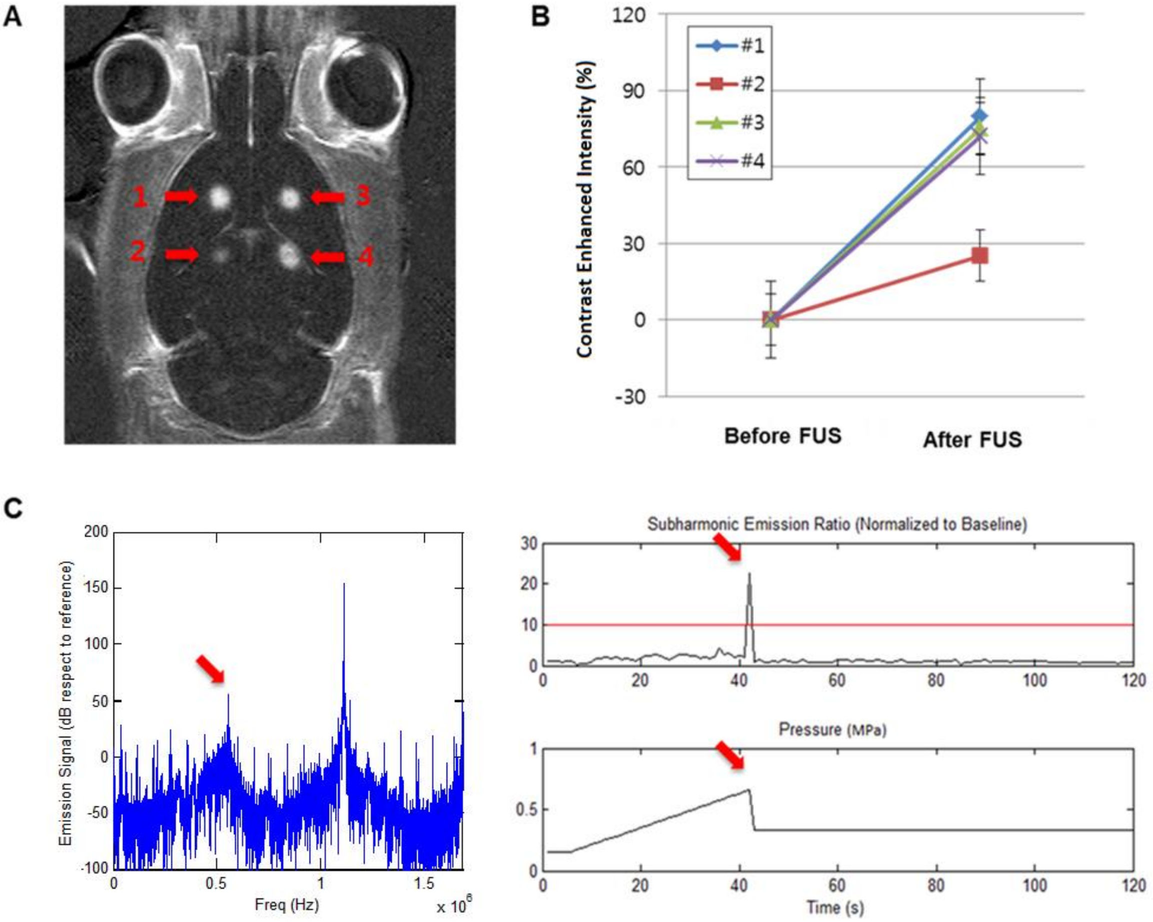


**Fig. S1:** BBB disruption results from the pilot study. (**A**) T1-weight contrast enhanced MR image showing four targets with different magnitude of BBB disruptions (**B)** MR contrast intensity increase at the target due to BBB disruptions. **(C)** Acoustic emission data acquired during sonication at target 1. The rise in subharmonic signal is noted with a red arrow (left), indicating active oscillation of microbubble. The acoustic pressure threshold was noted at the time of the rise in subharmonics (around 0.6 MPa).

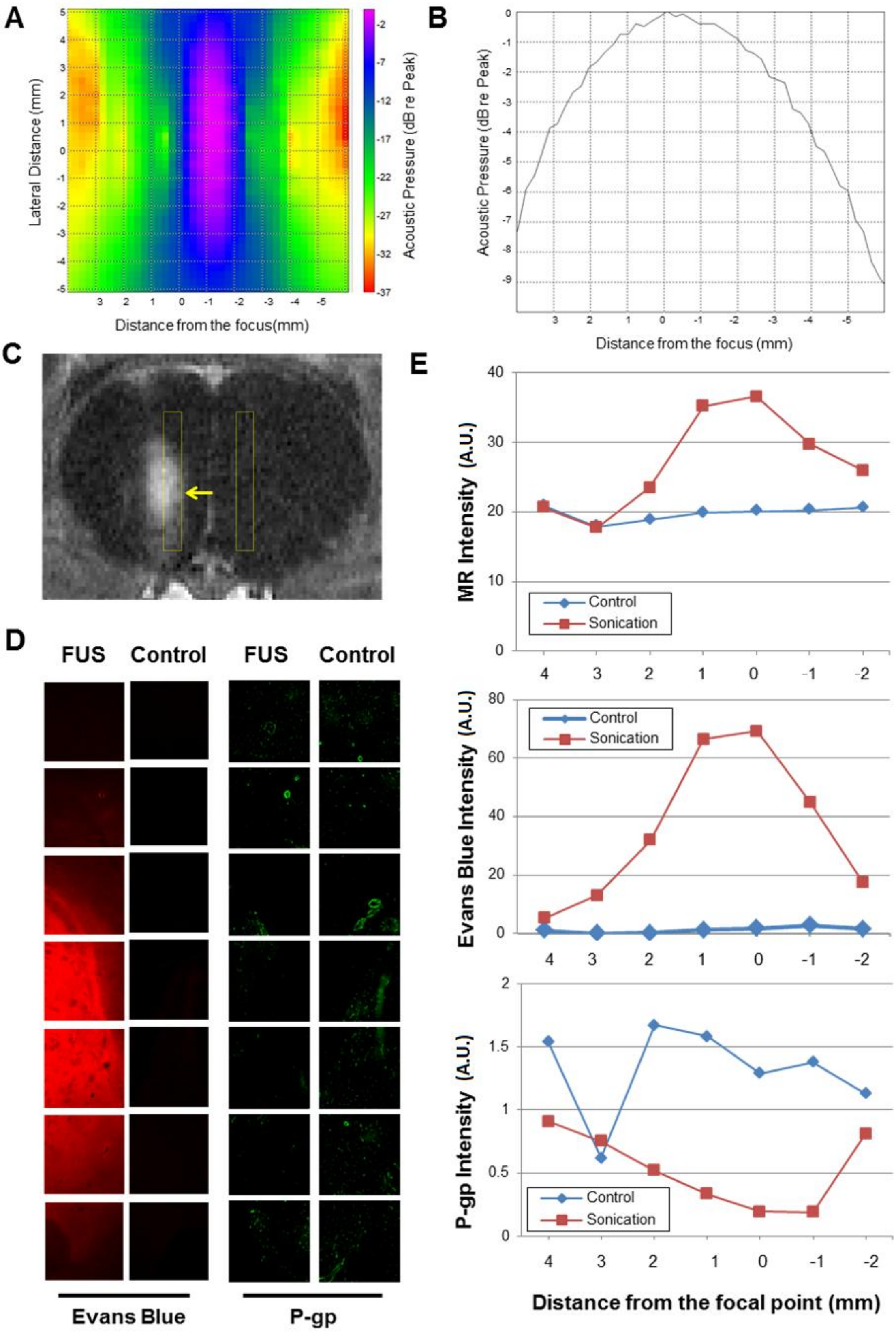
A
Lateral Distance (mm)
Distance from the focus(mm)
Acoustic Pressure (dB re Peak)
B
Acoustic Pressure (dB re Peak)
Distance from the focus (mm)
C
D
FUS
Control
FUS
Control
Evans Blue
P-gp
E
MR Intensity (A.U.)
Control
Sonication
Evans Blue Intensity (A.U.)
Control
Sonication
P-gp Intensity (A.U.)
Control
Sonication
Distance from the focal point (mm)

**Fig. S2:** (**A**) Acoustic pressure map generated from focused ultrasound. (**B**) Acoustic Pressure gradient along axial line around focal point (dB respect to peak pressure). (**C**) A Contrast enhanced T1-weighted MR Image of the rat brain at sonication region. Yellow squares indicate areas selected for fluorescence microscopy for P-gp expression and Evans Blue. Yellow arrow indicates the focal point of focused ultrasound. (**D**) Fluorescence microscopy images of the Evans Blue (Red) and the P-gp expression (Green). The sonication region (left column) and the control region (right column) are compared. (**E**) Line plot comparing the sonicated region and the control region along the axial direction (MR image contrast intensity, Evans Blue fluorescence intensity and P-gp fluorescence intensity, respectively)

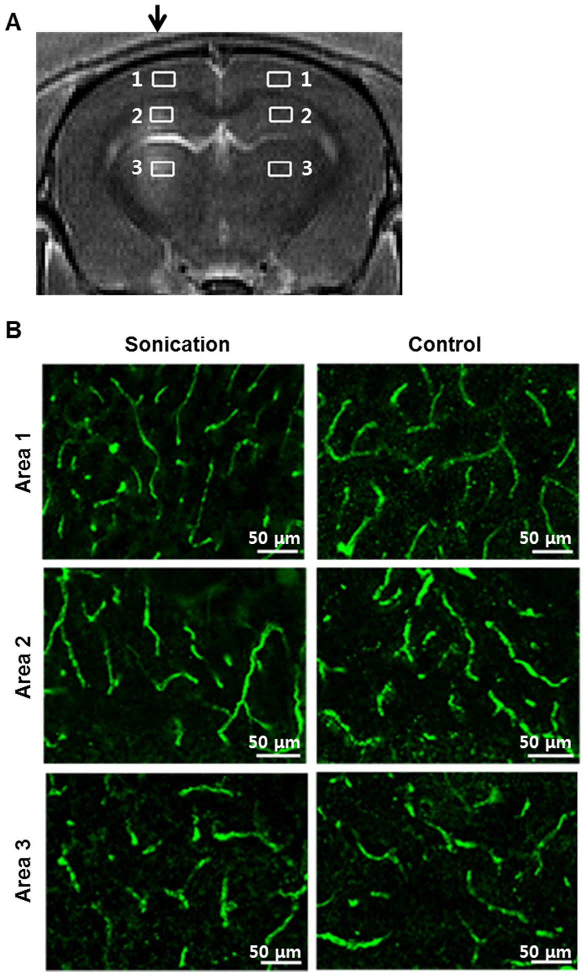


**Fig. S3:** RECA-1 expression at the sonicated region (**A**) A contrast enhanced T1-weighted MR image of a rat brain at sonication region (the opposite hemisphere of the brain served as a control). Areas indicated with white squares were selected for fluorescence spectroscopy analysis. (**B**) The fluorescence intensity at the sonicated region and the control region is shown to compare RECA-1 expression intensities (green)

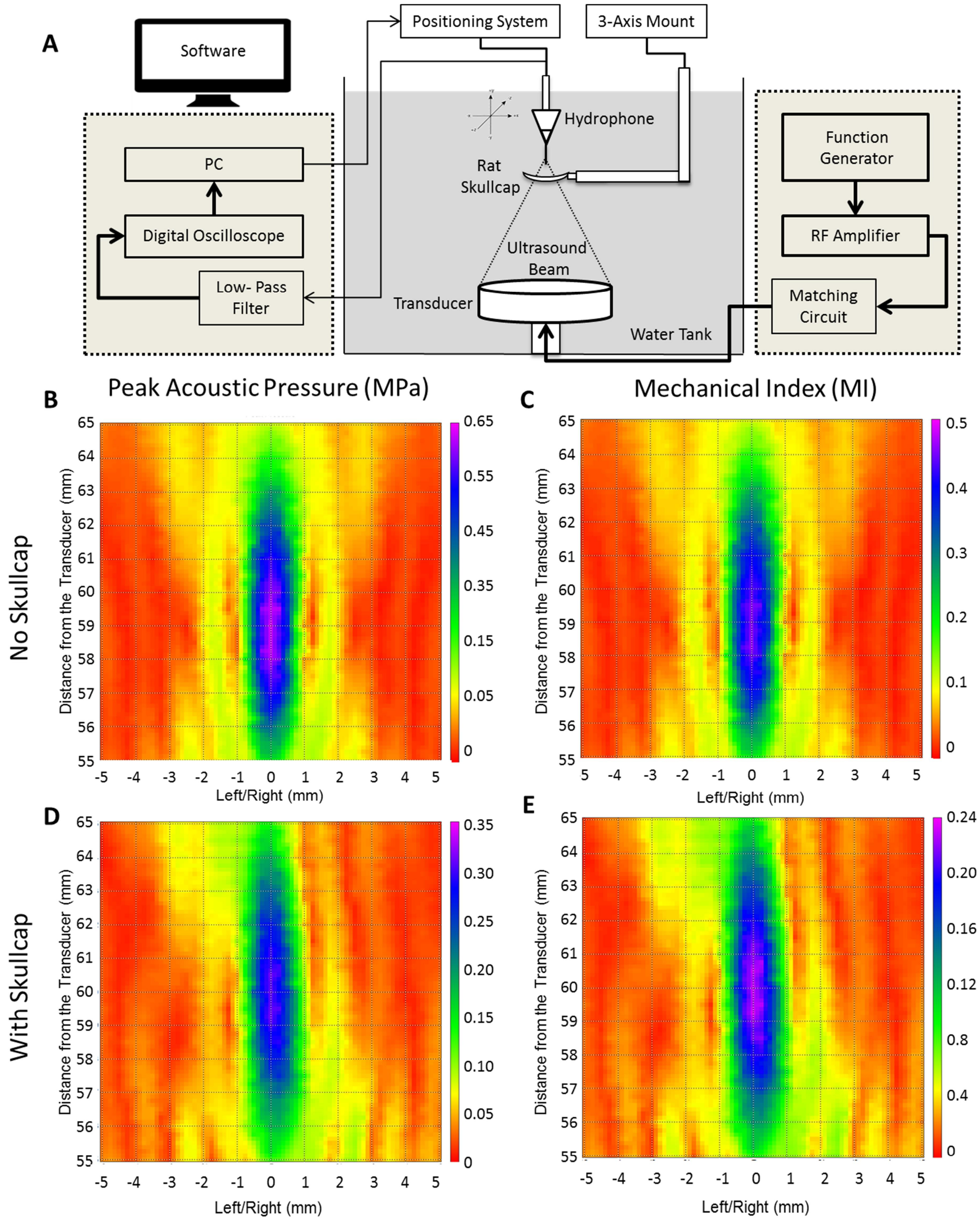


**Figure S4:** Measurement setup and acoustic output distribution at a focal zone **(A)** A schematic diagram of acoustic pressure distribution measurement with a rat skullcap **(B)** Peak acoustic pressure distribution without skull **(C)** Mechanical Index (MI) distribution without skull **(D)** Peak acoustic pressure distribution with a rat skullcap **(E)** MI distribution with a rat skullcap

**Table S1.** MR Imaging Parameters

| Sequence | Use | TR (ms) | TE (ms) | FOV ($mm^2$) | Matrix Size | Slice Thickness (mm) | NEX | ETL | Band-width (kHz) |
|---|---|---|---|---|---|---|---|---|---|
| **T1-weighted RARE (±Gd-DTPA)** | Detection of BBB Disruption | 1500 | 6.5 | 40×40 | 256×256 | 1.5 | 3 | 2 | ±15.6 |
| **T2-weighted RARE** | Treatment Planning, Detection of edema | 2500 | 33 | 40×40 | 256×256 | 1.5 | 2 | 2 | ±15.6 |
